# Pre-Exposure Intranasal Treatment with Neomycin Sulfate Reduces Transmission of Influenza B Virus

**DOI:** 10.3390/antibiotics15030245

**Published:** 2026-02-26

**Authors:** Mariia V. Sergeeva, Daria Shamakova, Kira Kudrya, Nikita Zagriadskii, Daria M. Karachevtseva, Aleksandr A. Matichin, Arman Muzhikyan, Marina Stukova

**Affiliations:** 1 Smorodintsev Research Institute of Influenza, The Ministry of Health of the Russian Federation, 197022 St Petersburg, Russia; 2 Home of Pharmacy, Research and Manufacturing Company (RMC), 188663 Kuzmolovskiy, Russia

**Keywords:** neomycin sulfate, interferon, host-directed antiviral, intranasal treatment, influenza B virus, transmission, guinea pigs, ferrets

## Abstract

**Background/Objectives**: Influenza B virus infection contributes substantially to annual morbidity and mortality, accounting for 20% to 30% of influenza-associated deaths worldwide. Although vaccination reduces the risk of severe disease, widely used inactivated influenza vaccines are often insufficient to prevent virus transmission. Moreover, influenza B viruses are less susceptible to commonly used antivirals than influenza A viruses. New approaches are therefore required to decrease disease burden and limit virus spread. Neomycin, an aminoglycoside antibiotic, was recently shown to mitigate SARS-CoV-2 transmission in a hamster model. Here, we conducted an exploratory study to assess the effect of neomycin on influenza B virus transmission. **Methods**: Contact transmission was evaluated using a guinea pig model (n = 4 per group), and aerosol transmission was assessed using a ferret model (n = 6 per group). Animals in the experimental groups received neomycin sulfate (5 mg/guinea pig, 20 mg/ferret) or placebo intranasally, starting one day before exposure to infected animals and continuing for four days thereafter. In the guinea pig study, an additional control group received intranasal interferon alpha. Viral transmission to contact animals was assessed by RT-PCR and virus culture of nasal washes collected over two weeks. Clinical signs and body weight were monitored daily. **Results**: In the guinea pig model, 75% of contact animals became infected with influenza B virus regardless of treatment. Neither neomycin nor interferon alpha prevented infection, although both delayed the onset of viral shedding in contact animals. In the ferret model, infection occurred in 33% of placebo-treated contact animals, whereas no viral shedding was detected in the neomycin-treated group. **Conclusions**: Prophylactic intranasal neomycin treatment has the potential to protect exposed individuals from aerosol transmission of influenza B virus during influenza outbreaks.

## 1. Introduction

Seasonal viral respiratory infections, especially influenza, contribute substantially to global morbidity and mortality. Each year, influenza B virus causes millions of infection cases, leading to respiratory complications, including bronchitis, pneumonia, and exacerbations of chronic conditions like asthma, particularly in children and the elderly [1]. Epidemiological studies estimate that influenza B virus is responsible for up to 20–30% of all influenza-related deaths [2]. Vaccination is an effective way to prevent severe disease, yet widely used inactivated influenza vaccines fail to reduce virus transmission and spread [3,4]. Antivirals are vital for those who have contraindications to vaccination or respond poorly to vaccines, as well as for treating infections early to prevent severe cases. Several studies reported that commonly used anti-influenza drugs, such as oseltamivir or baloxavir, may be less effective against influenza B viruses [5,6]. Therefore, new approaches are needed to decrease disease burden and limit virus spread.

Discovered in 1949, neomycin sulfate is an aminoglycoside antibiotic that has been approved for medical use since 1952. More than half a century later, new properties of neomycin were revealed, suggesting its potential as a host-directed antiviral. Neomycin was shown to independently induce expression of interferon-stimulated genes—the primary antiviral component of the innate immune system [7]. Earlier studies demonstrated various antiviral effects of neomycin in vitro and in vivo [8,9,10,11,12]. A recent paper by Mao et al. reported that intranasal neomycin provided protection against severe respiratory infection caused by influenza A virus and SARS-CoV-2 in an Mx1 congenic mouse model, and substantially mitigated contact transmission of SARS-CoV-2 in a hamster model [13].

Given the substantial contribution of influenza virus to the annual burden of acute respiratory viral infections and the promising antiviral properties of neomycin, we studied the influence of pre-exposure intranasal neomycin treatment on influenza B virus transmission using guinea pigs and ferrets as animal models. Guinea pigs represent a well-established small-animal model for contact influenza transmission [14,15,16] and, importantly, express a fully functional Mx protein [17], which is involved in the proposed neomycin-induced immune activation pathway [13]. Ferrets are considered the most relevant mammalian model for laboratory studies of influenza, as they share similarities with humans in lung physiology, cellular receptor distribution, and clinical signs of infection [18], and they also carry a functional Mx protein [19]. Moreover, influenza transmission studies in ferrets could provide meaningful predictions of secondary infection rates in humans [20].

## 2. Results

### 2.1. Effect of Neomycin Treatment on Influenza B Virus Contact Transmission in a Guinea Pig Model

#### 2.1.1. Intranasal Administration of Neomycin Sulfate Stimulates Mx Protein Expression in the Respiratory Tract of Guinea Pigs

Neomycin is thought to exert antiviral activity by activating innate immune factors, particularly the antiviral protein Mx [13]. We therefore first confirmed Mx expression in the respiratory tract of guinea pigs following intranasal neomycin administration. Animals were injected intranasally with 5 mg neomycin on day 0 and day 1, and Mx expression levels were assessed on day 2, i.e., 48 h after the first administration. Recombinant interferon alpha (IFNα) served as a control, as it has been shown to stimulate Mx protein synthesis in the lungs of guinea pigs 24–72 h after administration [17].

**Figure 1:**
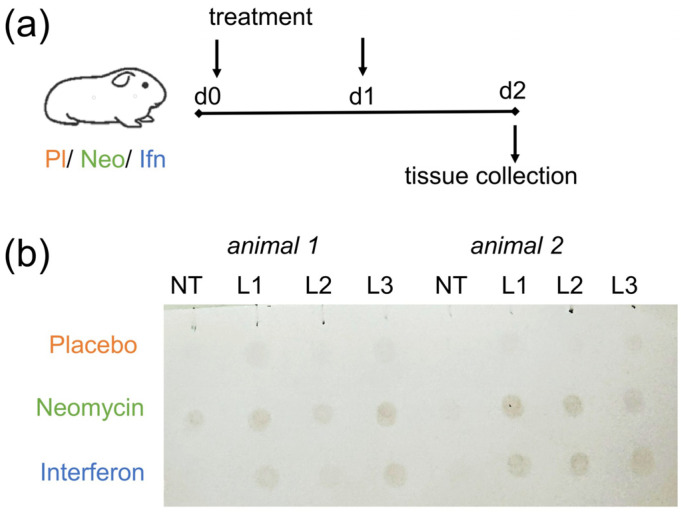
Mx protein expression in the respiratory tract of guinea pigs. (**a**) Experimental timeline. (**b**) Dot blot analyses of Mx expression in tissue homogenates. Representative results for two animals from each group are shown. Tissue type is indicated at the top: NT, nasal turbinates; L1–L3, lung lobes. The intensity of brown staining indicates relative Mx protein expression in the tissue.

Dot blot analysis revealed Mx protein expression in the lungs of all guinea pigs that received intranasal neomycin sulfate or IFNα twice at a one-day interval (Figure 1). In contrast, Mx expression in nasal turbinate tissue was detected in only one guinea pig treated with neomycin sulfate. These results confirm the ability of intranasal neomycin to activate innate immune factors in the respiratory tract.

#### 2.1.2. Pre-Exposure Neomycin Sulfate Treatment Delays Contact Influenza B Virus Infection

**Figure 2:**
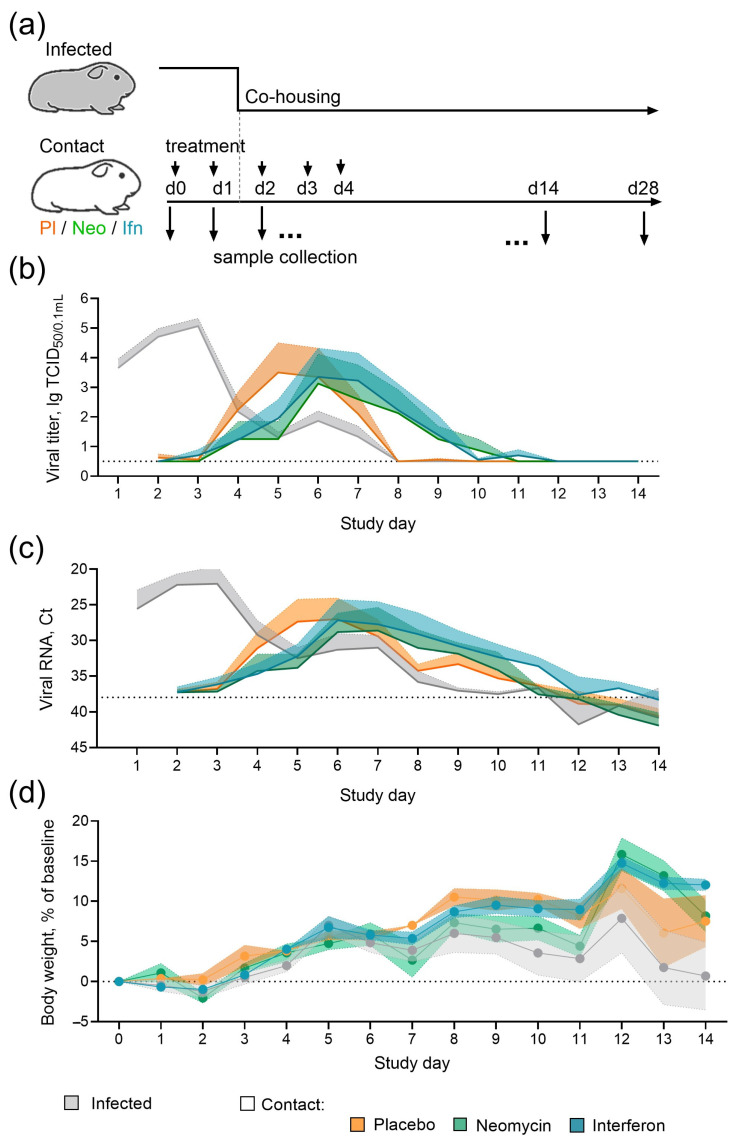
Effect of neomycin on influenza B virus transmission in guinea pigs. (**a**) Experimental timeline. (**b**) Infectious influenza virus titers in nasal swabs. (**c**) Influenza B virus RNA levels in nasal swab samples; Ct, real-time RT-PCR threshold cycle. Panels (**b**,**c**) show the dynamics of mean values with the upper range of the standard error of the mean (SEM; n = 6 for infected animals, n = 4 for contact animals). The dotted line indicates the detection limit. Individual data are presented in Appendix A Figure A1. (**d**) The dynamics of changes in guinea pig body weight, with mean values and SEM (n = 6 for infected, n = 4 for contacts).

Guinea pigs were chosen as a conventional small animal model of contact influenza transmission [14,15,16] to assess the ability of neomycin to reduce susceptibility to infection, similarly to recombinant IFNα [21] that served as a control in this experiment. Guinea pigs were pre-treated intranasally with neomycin, IFNα, or placebo and then placed in contact with infected animals (Figure 2a). During the 14-day contact period, two treated guinea pigs were co-housed with one infected animal (ratio 2:1). Animals from the infected group were inoculated intranasally with influenza B/Washington/1/2019 virus one day before the contact.

**Figure A2:**
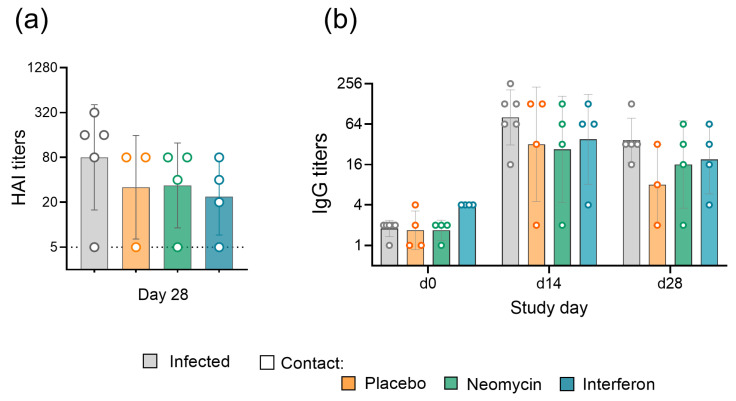
Antibody response in infected and contact guinea pigs. Individual values and geometric means (±SD) are presented. (**a**) Serum virus-specific antibody titers measured by hemagglutination inhibition assay. The dotted line indicates the detection limit. (**b**) Virus-specific IgG titers in nasal washes measured by ELISA. The dotted line indicates the geometric mean signal in naïve animals serving as the limit of detection.

By the end of the observation period, the efficiency of influenza B/Washington/1/2019 virus transmission from infected to contact guinea pigs reached 75%, regardless of the administered drug/placebo. In the placebo group, viral shedding peaked on days 5–6 of the study, with an average titer of 3.5 lgTCID_50_/0.1 mL. In the neomycin-treated group, viral shedding peaked one day later, on day 6, and the average virus titer was lower, reaching 3.1 lgTCID_50_/0.1 mL. IFNα treatment also delayed peak viral shedding by one day (Figure 2b). *Post hoc* statistical analyses confirmed a significant effect of the time-treatment interaction on viral titers (mixed-effects model REML, *p* = 0.0024). However, pairwise comparisons at individual time points were not significant (Dunnett’s test, *p* > 0.05). RT-PCR analysis of the viral load generally corresponded to the titration results, indicating delayed viral shedding in neomycin and interferon-treated groups (Figure 2c). Humoral immune response assessment revealed 100% seroconversion in infected animals and 75% seroconversion in contact groups (Appendix A, Figure A2), and the same percentage of animals developed local virus-specific IgG antibody responses. A slight trend toward higher antibody levels in infected animals compared with contact animals was observed, but the differences were not statistically significant. Throughout the experiment, all groups showed positive changes in body weight, suggesting that the applied neomycin regimen and dose were well tolerated (Figure 2d).

### 2.2. Pre-Exposure Intranasal Neomycin Sulfate Treatment Prevents Aerosol Transmission of Influenza B Virus Infection in a Ferret Model

**Figure 3:**
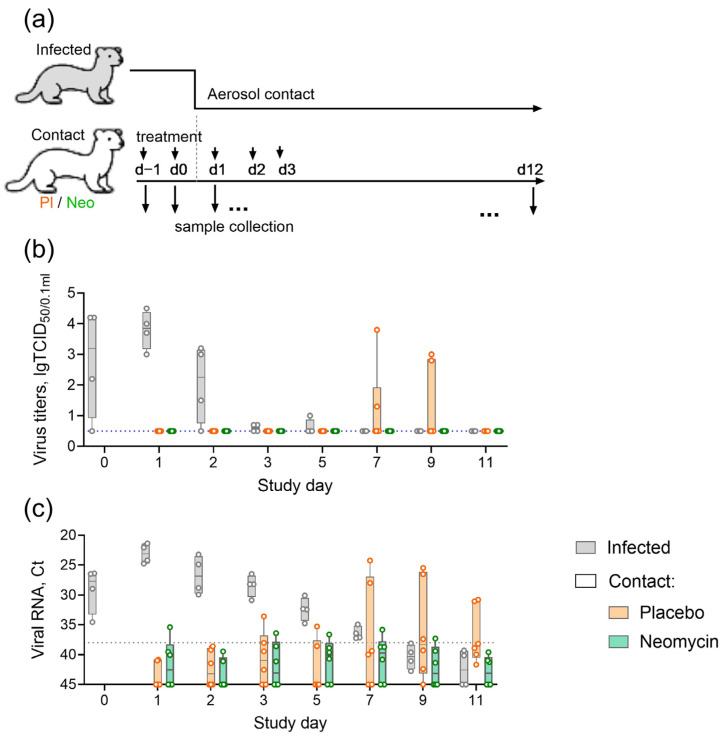
Effect of neomycin on influenza B virus transmission in ferrets. (**a**) Experimental timeline. (**b**) Infectious influenza virus titers in nasal wash samples. (**c**) Influenza B virus RNA in nasal wash (NW) samples; Ct, real-time RT-PCR threshold cycle. Individual values and summary statistics are presented in boxplots. The dotted line indicates the detection limit.

Ferrets are highly susceptible to infection with non-adapted human influenza viruses, which makes them a widely used model in virus transmission experiments [18,22,23]. In this study, we evaluated the ability of neomycin to reduce the risk of influenza infection by the aerosol route. Ferrets were pre-treated intranasally with neomycin or placebo and then placed in contact with infected animals (Figure 3a). During the contact period, three neomycin-treated, three placebo-treated, and two infected animals (ratio 3:3:2) were housed in a single room with natural ventilation, each in individual cages separated by approximately 1 m to simulate aerosol virus spread. One day before contact, the infected group was inoculated intranasally with influenza B/Brisbane/60/2008 virus, which has previously been shown to transmit effectively in ferrets [23].

Within the 11-day observation period, infected animals showed viral shedding from day 0 to day 5, with a peak observed on day 1 (48 h after infection). In the placebo group, viral shedding was detected in 2/6 ferrets (33%) between days 7 and 9. In the neomycin group, no cases of infectious viral shedding were recorded (Figure 3b) (*post hoc* Fisher’s test *p* = 0.45). Importantly, although no live virus was detected, nasal swabs from this group and the placebo group were periodically tested positive for influenza viral RNA by real-time RT-PCR (Figure 3c). Overall, 3/6 animals in the placebo group and 4/6 animals in the neomycin group were PCR-positive for influenza B virus at any time point. In the placebo group, 2/3 PCR-positive animals (66%) developed influenza infection, whereas none of the PCR-positive animals in the neomycin group developed infection (*post hoc* Fisher’s test, *p* = 0.14). By the end of the study (day 11), seroconversion was observed only in animals from the infected group (Supplementary Figure S1).

**Figure 4:**
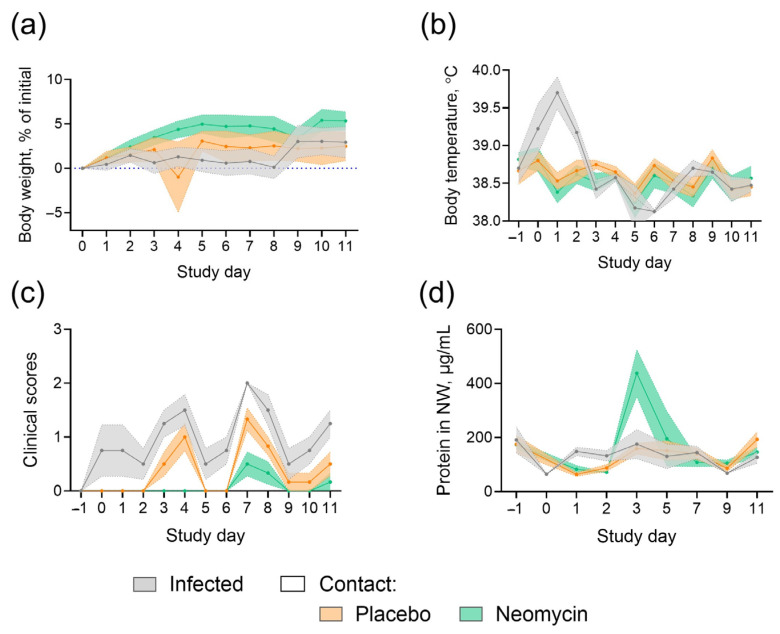
Clinical monitoring of ferrets during influenza B transmission study. (**a**) Body weight dynamics. (**b**) Body temperature. (**c**) Summary clinical score. (**d**) Total protein in nasal wash samples. Mean values and SEM are shown (n = 4 for infected animals, n = 6 for contact animals).

Throughout the study, nasal discharge or sneezing was occasionally observed in ferrets from all groups, while no signs of diarrhea or decreased activity were detected. Weight loss and fever occurred only in infected animals (Figure 4a,b), indicating milder disease in placebo-treated animals that acquired infection by contact. No significant pathological changes were detected in ferret lung tissue (Supplementary Figure S2). An increase in protein content was observed in neomycin-treated ferrets on day 3 (the day of the last drug administration; Figure 4d).

## 3. Discussion

The urgent need for broad-spectrum antiviral drugs persists due to the continued circulation of viruses with high mutational variability, such as influenza, and was recently highlighted by the COVID-19 pandemic. Rapid influenza virus evolution continues to generate antiviral-resistant mutants and facilitates their spread. In this context, host-directed antiviral therapies represent a more sustainable alternative. Recent research suggesting antiviral activity of the antibiotic neomycin [13] motivated the present study, which evaluated neomycin as a pre-exposure treatment intended to reduce the risk of influenza infection across different transmission routes.

Using a guinea pig model of contact influenza transmission, we demonstrated that intranasal neomycin treatment did not prevent influenza B infection. Still, viral shedding was delayed in neomycin-treated contact animals relative to placebo controls. A similar pattern was observed previously in a highly susceptible hamster model of SARS-CoV-2 infection, where neomycin did not prevent infection but reduced viral load [13].

Our study also included IFNα as a reference treatment, given earlier reports of effective prevention of contact transmission of influenza A/H1N1, A/H5N1, and A/H1N1pdm09 viruses in guinea pigs [17,21]. Although several studies showed efficient transmission of influenza B virus in this model [16,24,25], interferon activity against influenza B has not been explored. Here, we demonstrated that IFNα failed to fully prevent influenza B virus transmission despite the induction of Mx protein expression, even though IFN treatment delayed peak viral shedding in contact animals, similar to neomycin. The observed difference in sensitivity to interferon-stimulated responses between influenza A and B viruses could be associated with an enhanced ability of influenza B to suppress host cytokine signaling, as shown recently in vivo in ferrets and in vitro in primary ferret nasal epithelial cultures [26,27,28], in contrast to earlier findings from immortalized cell lines [29].

Households are important sites of influenza transmission, and aerosol spread accounts for approximately half of all transmission events [30,31]. Therefore, in the second part of the study, we attempted to explore neomycin activity against aerosol-transmitted infection in a ferret model. Ferrets are highly susceptible to infection with non-adapted human influenza viruses and, along with guinea pigs, are widely used in virus transmission studies [18,22]. Importantly, ferrets carry a functional Mx protein [19], and intravenous treatment with a RIG-I agonist was associated with upregulation of interferon-stimulated genes in ferrets, reducing disease severity in a contact transmission setting [32]. In a previous study of baloxavir, influenza B virus transmission occurred in all contact ferrets under both direct and aerosol exposure, with the drug only reducing viral load in contact animals [23]. In the present study, aerosol transmission occurred in 33% of placebo-treated contact ferrets, and no viral shedding was detected in the neomycin-treated group. During the observation period, nasal swabs from 66% of neomycin-treated ferrets were PCR-positive for viral RNA, but these animals were negative for infectious virus and did not develop influenza. By contrast, under the same conditions, two PCR-positive placebo-treated ferrets developed infection. These findings are consistent with viral entry into the nasal cavity of contact ferrets and support a potential role for neomycin in preventing the onset of productive infection.

The main limitations of this study are the small number of animals and its exploratory design. Group sizes of 4 to 6 animals are conventional in influenza transmission experiments [18,33] and could not be exceeded in an exploratory study for ethical reasons. However, the present study was underpowered because transmission of the Brisbane/60/2008 virus in placebo-treated ferrets (33%) was lower than previously reported (100%) [23]. When analysis was restricted to PCR-positive contact ferrets, the calculated statistical power was 49%. At the same time, the observed contact infection rate aligns well with reported secondary infection rates of 20–50% in household contacts [34,35,36]. As real-world transmission risk is generally lower, we assume that the model used here may better reflect natural exposure conditions. Confirmation of statistical significance will require replication using larger sample sizes. Further studies with larger sample size are important to enable statistically robust conclusions.

Another limitation is the absence of detectable antibody responses in contact ferrets, including those that were PCR-positive or TCID50-positive. This could be attributed to insufficient immune stimulation due to low viral exposure in the absence of productive infection, or to the short interval between the onset of infection and the final blood and nasal wash sampling. Robust HAI antibody responses in ferrets are typically detected from 7 days post influenza A infection [37], and local antibody responses are not usually detectable before 5–6 dpi [38]. These responses may be further delayed following influenza B virus infection [28]. In our study, infection in placebo-treated contact animals began on day 7, but the observation period ended on day 11, likely providing insufficient time for detectable antibody development. In addition, assessment of local antibody responses was limited to IgG because of the unavailability of anti-ferret IgA secondary antibodies; nevertheless, published data suggest comparable dynamics of IgG and IgA responses in the ferret upper respiratory tract after influenza infection [38,39].

Since neomycin sulfate is highly toxic when administered intraperitoneally and alters gastrointestinal microbiota when given orally [40], its dosage and treatment regimen should be considered. In this study, body-weight-adjusted dosing ranged between 10 and 15 mg/kg/day across animal models, remaining within permissible veterinary limits for neomycin sulfate administration. Intranasal delivery in small volumes (50 μL per guinea pig and 200 μL per ferret) complied with international recommendations for these species [41] and helped restrict drug exposure to the respiratory tract. However, given the high rate of mucociliary clearance [42], partial drug passage into the gastrointestinal tract could not be excluded, and we also monitored the general health status of the animals throughout the study. No adverse clinical signs or body-weight changes were observed in neomycin-treated animals during the monitoring period. The only detectable difference was an increase in total protein content in nasal washes from neomycin-treated ferrets, which may reflect a transient local inflammatory response, as previously reported in influenza-infected animals [43]. Although previous research suggested immune suppression after neomycin treatment [44], immune responses to infection were preserved in our study, as indicated by systemic serum HAI antibody levels and local IgG production in neomycin-treated guinea pigs. This is likely attributable to differences in dosing and administration regimens.

Despite these limitations, our findings suggest that short-term intranasal neomycin treatment was well-tolerated, induced Mx antiviral protein expression in the respiratory tract, and increased resistance to influenza B virus infection in both guinea pigs and ferrets.

## 4. Materials and Methods

### 4.1. Pharmaceuticals

Lyophilized neomycin sulfate (CJCS Agropharm, Voronezh, Russia), approved for veterinary use in the Russian Federation and containing 680 μg of active substance per mg of powder lyophilizate, was diluted in sterile water to a concentration of 100 mg/mL of active substance immediately before administration.

Lyophilized human recombinant IFNα (Reaferon-ES, JSC Vector-Medica, Kol’tsovo, Russia), approved for human use in the Russian Federation and containing 3,000,000 U/ampule, was diluted in sterile water to a concentration of 5,000,000 U/mL immediately before administration.

### 4.2. Animals

Female guinea pigs (2–3 months old) were obtained from the Rappolovo breeding facility of the NRC ‘Kurchatov Institute’ (Leningrad region, Russia) and quarantined for two weeks. Only healthy animals with body weight within ±10% of the mean were included in the study, and no animals were excluded. All animals were confirmed to be seronegative for influenza B virus by hemagglutination inhibition assay. Guinea pigs were randomly assigned to experimental and control groups. Each animal was assigned with the random number generated in MS Excel software (Microsoft, Redmond, WA, USA) using RAND function. The generated numbers were then sorted in ascending order. The resulting list was used to sequentially include animals in experimental groups from first to last. Group allocation was not blinded at any stage of the experiment.

Male ferrets (7–8 months old) were obtained from a breeding farm ‘Novye Mekha’ (Tver, Russia) and quarantined for three weeks. All animals were confirmed to be seronegative for influenza B virus by hemagglutination inhibition (HAI) assay. Only healthy ferrets were included in the study, and no animals were excluded. Given the small number of animals (n = 16) and high inter-individual variability, ferrets were stratified by weight (into 5 strata) and then assigned to the three experimental and control groups to avoid significant differences in mean body weight between groups. Veterinarians and technicians, but not the researchers, were blinded to group allocation throughout the experiment. Only blinded staff performed any animal procedures, including clinical signs assessment.

All experiments were conducted in accordance with the European Convention for the Protection of Vertebrate Animals used for Experimental and other Scientific Purposes (ETS No. 123) and were approved by the institutional bioethics committee of the Smorodintsev Research Institute of Influenza (Protocol #15 dated 30 June 2025) and the Local Ethics Committee of the RMC ‘Home of Pharmacy’ (Approval No. 1.47/25 dated 12 November 2025).

### 4.3. Viruses

Influenza B/Brisbane/60/2008 and B/Washington/02/2019 viruses were obtained from the Centers for Disease Control and Prevention (Atlanta, GA, USA), WHO Collaborating Centers for Reference and Research on Influenza as a part of the GISRS program for the National Influenza Centre at the Smorodintsev Research Institute of Influenza. All strains were propagated in the allantoic cavity of 10-day-old chicken embryos at 32 °C for 48 h. Virus infectious activity in chicken embryos (EID_50_) was estimated by the standard titration procedure and calculated by the Reed and Muench method [45].

### 4.4. Exploring of Mx Protein Expression in Guinea Pigs

The study included 6 animals (n = 2 per group). Animals in the neomycin group were administered 5 mg/animal/day neomycin sulfate (ZAO Agropharm, Voronezh, Russia). Animals in the negative control (placebo) group received phosphate-buffered saline (PBS; Biolot, Saint-Petersburg, Russia). Animals in the positive control group were administered 250,000 U/animal/day of IFNα (AO Vector-Medica, Kol’tsovo, Russia). The drugs/placebo were administered intranasally on day 0 and day 1, at a volume of 50 μL per animal. On day 2, all animals were euthanized, and nasal turbinates (NT) and lung lobe tissue (L1–L3) were collected. Tissue samples were homogenized in PBS using 3 mm steel beads and a Tissue Lyser homogeniser (Qiagen, Germantown, MD, USA) for the subsequent dot blot analysis of Mx expression.

### 4.5. Dot Blot Assay

Tissue homogenates were clarified by centrifugation at 7000 *g* for 10 min and diluted 1:10 in sterile water. Total protein content was measured using the QuDye Protein reagent kit (Lumiprob RUS, Moscow, Russia) and a Qubit fluorometer (Thermo Scientific, Waltham, MA, USA). Samples were adjusted for total protein concentration, mixed with 4× Laemmli buffer with β-mercaptoethanol (Biolabmix, Novosibirsk, Russia), boiled for 7 min, and cooled on ice. Denatured samples (2 μL) were applied to a nitrocellulose membrane (Servicebio, Wuhan, China) and left to dry completely. The membrane was blocked overnight at room temperature in PBS containing 5% non-fat dry milk (Stoing, St. Petersburg, Russia) and 0.1% TWEEN20 (NeoFroxx, Einhausen, Germany). It was then incubated overnight at room temperature with mouse anti-Mx1 antibodies (#CTX84067, GeneTex, Irvine, CA, USA) diluted 1:200 in blocking buffer. After three washes with PBS containing 0.1% TWEEN20, the membrane was incubated for 1 h at room temperature with horseradish peroxidase-conjugated secondary anti-mouse antibodies (ab97023, Abcam, Cambridge, UK) diluted 1:1000 in blocking buffer. After three washes, the membrane was incubated in PBS containing 0.05% DAB substrate (Serva, Heidelberg, Germany) with 0.015% hydrogen peroxide (Vekton, St. Petersburg, Russia) until color developed (for about 30 min). The membrane was then rinsed with distilled water and imaged.

### 4.6. Influenza Transmission in a Guinea Pig Model

Guinea pigs (n = 4 per group) were treated intranasally with PBS (Biolot, St Petersburg, Russia), 5 mg of neomycin sulfate (ZAO Agropharm, Russia), or 250,000 U of IFNα (AO Vector-Medica, Russia) in a total volume of 50 μL per animal on study days 0, 1, 2, 3, and 4. Another group of guinea pigs (n = 6) was infected with 5 lg EID_50_ of B/Washington/02/2019 virus in a volume of 20 μL on day 0 in a separate facility. Treated and infected guinea pigs were placed in contact on day 1 and co-housed at a ratio of 1:2 (infected:contact) for the subsequent 14 days. Animals were monitored daily for changes in body weight and clinical signs, including nasal discharge, decreased activity, and diarrhea. Nasal washes were collected daily by rinsing the nasal cavity with 1 mL PBS into a Petri dish. Viral load in nasal washes was assessed by TCID_50_ assay and real-time RT-PCR. Serum samples and nasal washes for antibody detection by hemagglutination inhibition (HAI) assay and ELISA, respectively, were collected on day 0 (before all manipulations) and on day 28.

### 4.7. Influenza Transmission in a Ferret Model

Ferrets (n = 6 per group) were treated intranasally with PBS (Biolot, St Petersburg, Russia) or 20 mg of neomycin sulfate (ZAO Agropharm, Russia) in a total volume of 200 μL per animal on study days −1, 0, 1, 2, and 3. Another group of ferrets (n = 4) was infected with 6 lg EID_50_ of B/Brisbane/60/2008 virus in a volume of 200 μL on day −1 in a separate facility. Treated and infected ferrets were placed in contact on day 1 and housed in separate cages within the same room at a ratio of 2:3:3 (infected:contact placebo:contact neomycin) for the subsequent 11 days. Animals were monitored daily for changes in body weight and clinical signs, including sneezing, nasal discharge, decreased activity, and diarrhea. Nasal washes were collected by rinsing the nasal cavity with 1 mL PBS into a Petri dish. Viral load in nasal washes was assessed by TCID_50_ assay and real-time RT-PCR. Serum samples and nasal washes for antibody detection by hemagglutination inhibition (HAI) assay and ELISA, respectively, were collected on day—1 (before all manipulations) and on day 12.

### 4.8. Virus Infectious Activity Measurement

Virus infectious activity was determined by titration of clarified nasal wash samples in MDCK cell culture (#FR-58, International Reagent Resource). The assay was performed in 96-well culture plates (TPP, Trasadingen, Switzerland). Cells were infected by adding 100 µL of 10-fold serial dilutions of virus-containing material (4 wells per dilution) and incubated for 5 days at 34 °C in 5% CO_2_. Results were evaluated by hemagglutination of culture media with 0.5% chicken erythrocytes. The 50% tissue culture infectious dose (TCID_50_) was calculated using the method of Reed and Muench [36]. Viral titers were expressed as lg TCID_50_/0.1 ml.

### 4.9. Real-Time RT-PCR

RNA was extracted from nasal wash samples using the MagnoPrime FAST-R kit (NextBio, Moscow, Russia) according to the manufacturer’s instructions. Single-step real-time RT-PCR was performed using the BioMaster RT-qPCR (2×) kit (Biolabmix, Novosibirsk, Russia) following the manufacturer’s protocol. Viral RNA was detected with CDC primers and probes targeting the influenza B NS gene [46].

### 4.10. Antibody Detection by Hemagglutination Inhibition (HAI) Assay

Sera were pretreated 1:3 with RDE (Denka Seiken, Tokyo, Japan) at 37 °C for 19 h, heat-inactivated at 56 °C for 1 h, and then incubated with an equal volume of 10% chicken red blood cells at 2–8 °C for 1 h to remove natural agglutinins. Serial twofold dilutions of the treated serum samples were prepared in 96-well U-bottom plates with 25 μL of PBS per well. Viral antigen (25 μL/well) containing 4 hemagglutination units was added and incubated for 1 h. Next, 50 μL of 0.5% chicken red blood cells were added and incubated for 1 h at room temperature. Antibody titers were defined as the highest serum dilution that inhibited erythrocyte agglutination.

### 4.11. Enzyme-Linked Immunoassay (ELISA)

Nasal wash samples were analyzed for virus-specific IgG antibodies by ELISA. 96-well plates (Greiner Bio One, Monroe, NC, USA) were covered with 500 ng/well of the corresponding purified virus and blocked with 5% non-fat milk (Stoing, Russia) in PBS containing 0.1% TWEEN20 (NeoFroxx, Germany). Samples were two-fold serially diluted in blocking buffer, added to the wells, and incubated for 1.5 h at room temperature. Plates were washed with 0.1% TWEEN20 PBS and incubated with secondary antibodies, Anti-Guinea Pig IgG (whole molecule) Peroxidase (A5545, Sigma, St. Louis, MO, USA) or Goat Anti-Ferret IgG H&L (HRP) (ab112770, Abcam, Cambridge, UK), diluted 1:4000 in blocking buffer. After a final wash, the plates were developed with TMB substrate solution (Hema, Moscow, Russia) for 15 min at room temperature in the dark. The reaction was stopped by adding 2 M H_2_SO_4_ (Vekton, Russia). Optical density was measured at 450 nm using a Multiskan Skyhigh reader (Life Technologies, Carlsbad, CA, USA). Titers were calculated as the highest sample dilution producing a specific signal optical density above the threshold value of 0.100.

### 4.12. Histopathology Analysis

Lungs were fixed in 10% neutral buffered formalin for 24 h and processed using standard histological methods. 3–5 µm sections were stained with hematoxylin and eosin and imaged with a Carl Zeiss AxioScope 2 Plus microscope equipped with an AxioCam ERc5s camera and the AxioVision Rel software 4.8 (Carl Zeiss, Oberkochen, Germany). Lung tissue compartments (bronchioli, blood vessels, interstitium, and alveoli) were assessed for the severity of inflammation and focal accumulation of infiltrating cells.

### 4.13. Statistical Data Analysis

Raw data were imported into Prism v10.4.0 (GraphPad, San Diego, CA, USA). The following descriptive statistics were used: geometric mean, standard deviation (SD), arithmetic mean, and the standard error of the mean (SEM). The study was exploratory and, therefore, was not designed to test statistical hypotheses. Group sizes were determined based on previously published studies on influenza transmission [18,33,47]. *Post hoc* statistical analyses comparing placebo and treatment groups included a REML mixed-effect model with Dunnett’s *post hoc* test to assess differences in viral load in the guinea pig study (not-infected animals were excluded); and Fisher’s exact test to compare proportions of infected animals in the ferret study. Both analyses were performed using Prism v10.4.0 (GraphPad, USA).

## 5. Conclusions

Our findings support the potential repurposing of neomycin, an aminoglycoside antibiotic, as a host-directed antiviral. Neomycin was shown to reduce infection in the most relevant animal models of contact influenza transmission. Intranasal neomycin treatment delayed the onset of infection following close contact and reduced airborne transmission. Thus, prophylactic intranasal neomycin treatment may protect exposed individuals from aerosol transmission of influenza virus during outbreaks.

## Figures and Tables

**Figure A1:**
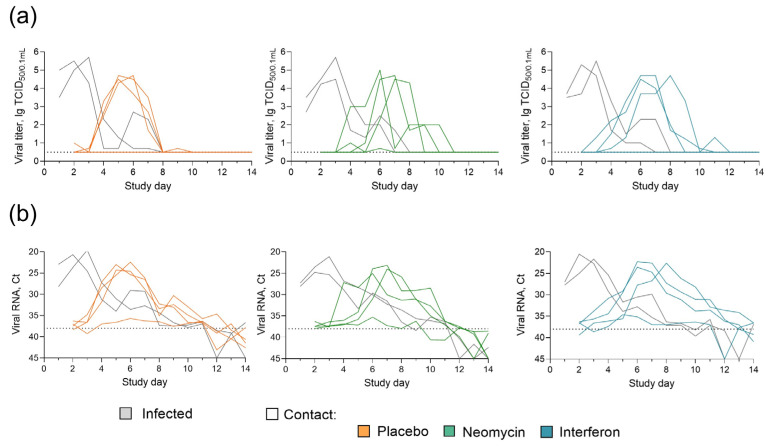
Effect of neomycin on influenza B virus transmission in guinea pigs (individual data). (**a**) Infectious influenza virus titers in nasal washes. (**b**) Influenza B virus RNA in nasal wash samples. Data from each guinea pig are shown by a separate line. The dotted line indicates the detection limit.

## Data Availability

The key data, including individual values, are presented in the manuscript or Supplementary Files. The additional raw data could be provided upon reasonable request to the corresponding author.

## Supplementary Materials

The following supporting information can be downloaded at https://www.mdpi.com/article/10.3390/antibiotics15030245/s1. Figure S1. Antibody response in infected and contact ferrets; Figure S2. Histopathological analyses of ferret lung tissue. References [48,49,50,51,52,53] are cited in the Supplementary Materials.

## Abbreviations

The following abbreviations are used in this manuscript:

EID_50_	50% embryonic infectious dose
ELISA	Enzyme-linked immunosorbent assay
U	Units of activity (for recombinant interferon alpha)
Mx	Myxovirus Resistance Protein
NT	Nasal turbinates
PBS	Phosphate-buffered saline
RT-PCR	Reverse transcription followed by polymerase chain reaction
SD	Standard deviation
SEM	Standard error of the mean
TCID_50_	50% tissue culture infectious dose
