# Supplementary material for: Pre-Exposure Intranasal Treatment with Neomycin Sulfate Reduces Transmission of Influenza B Virus
Source: Antibiotics (Basel). 2026 Feb 26;15(3):245. doi: 10.3390/antibiotics15030245 (PMC13024608; doi:10.3390/antibiotics15030245)
Supplement: Supplementary file 1 [file antibiotics-15-00245-s001.zip › Supplementary figures.pdf]

Supplementary figures

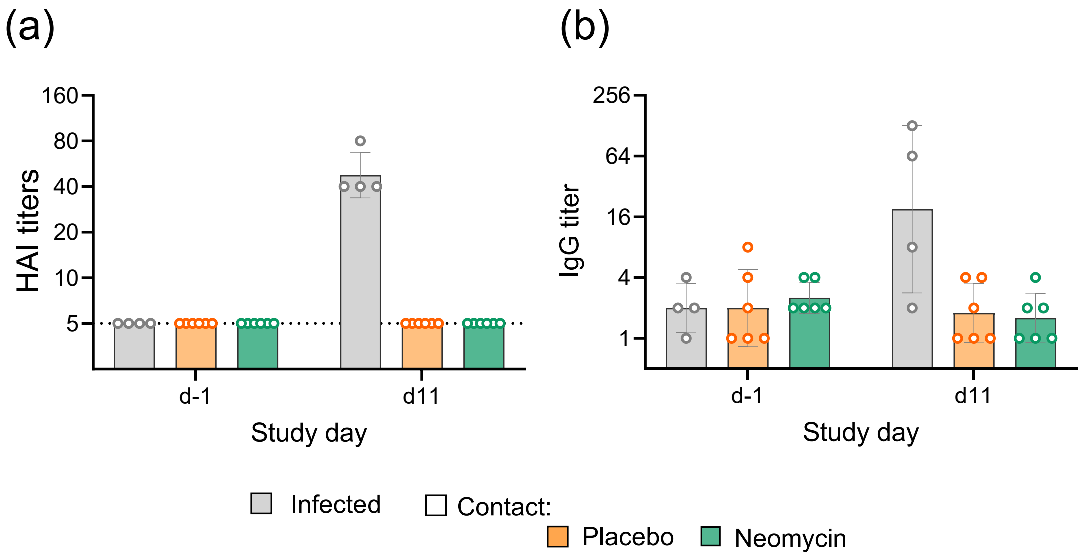

Figure S1 – Antibody response in infected and contact ferrets. Individual values and geometric means (±SD) are presented. (**a**) Serum virus specific antibody titres, measured in hemagglutination inhibition assay. Dotted line represents the limit of detection; (**b**) Nasal wash virus specific IgG titre measured in ELISA.

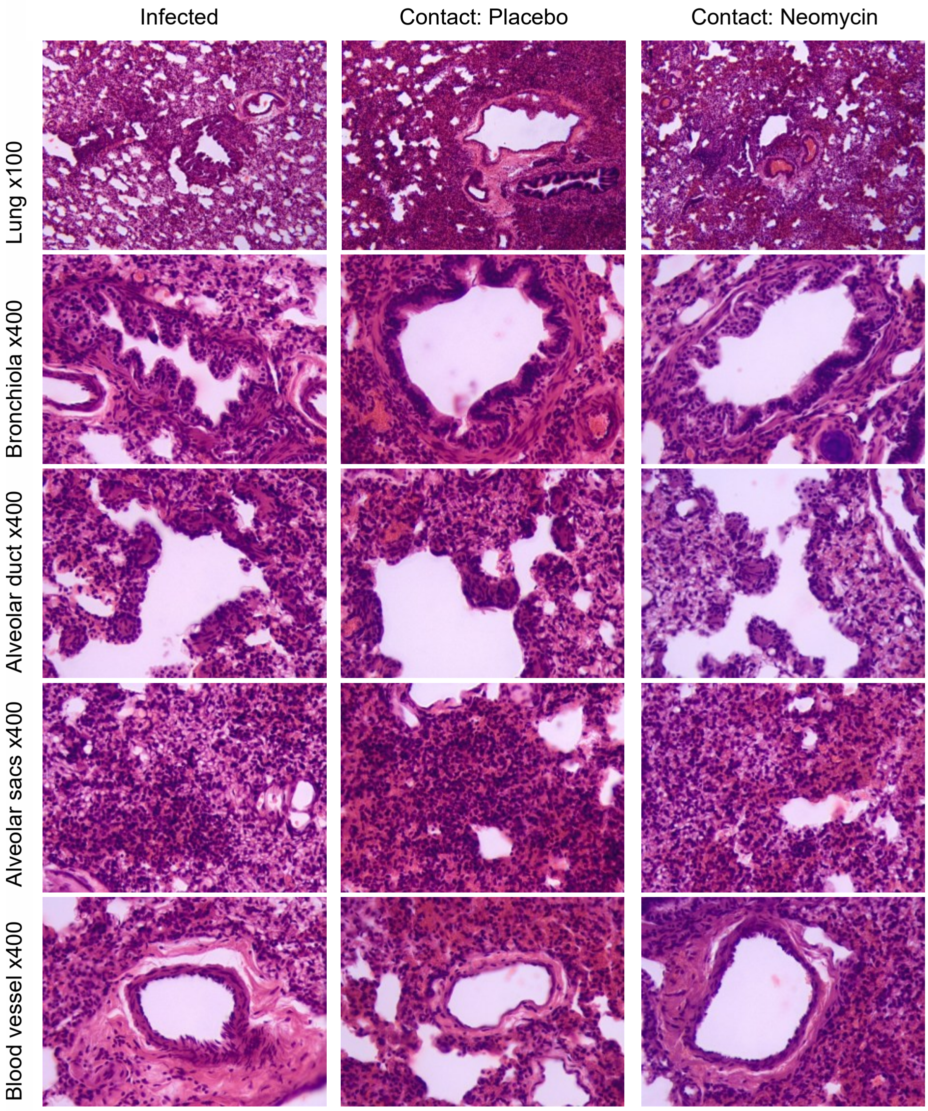

Figure S2 – Histopathological analyses of the lung tissue of ferrets. Representative microphotographs for each group and lung tissue elements are shown.
