# Supplementary figures and images for: Pre-Exposure Intranasal Treatment with Neomycin Sulfate Reduces Transmission of Influenza B Virus
Source: Antibiotics (Basel). 2026 Feb 26;15(3):245. doi: 10.3390/antibiotics15030245 (PMC13024608; doi:10.3390/antibiotics15030245)

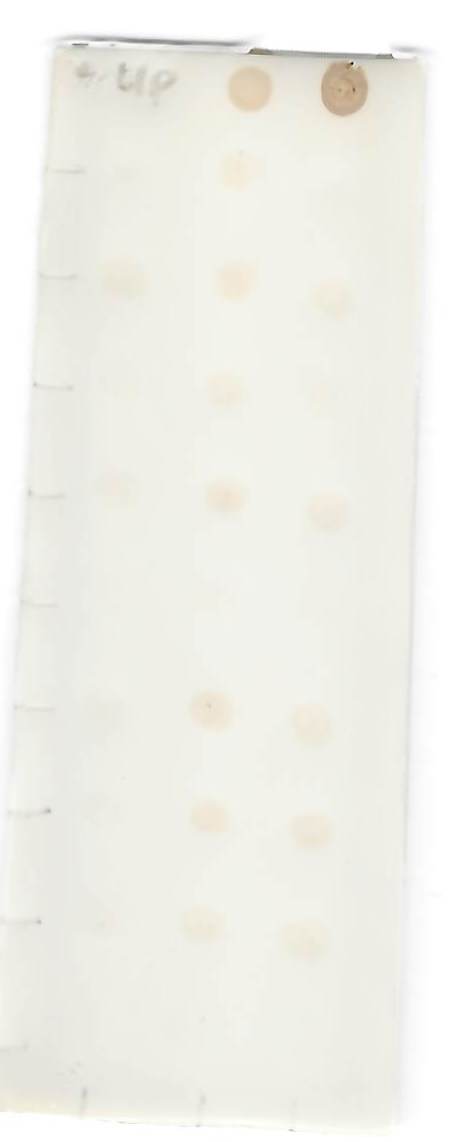

Supplement: Supplementary file 1 [file antibiotics-15-00245-s001.zip › Dot blot original picture scan.tif]
